# Federated Learning for Cardiovascular Disease Prediction: A Comparative Review of Biosignal- and EHR-Based Approaches

**DOI:** 10.3390/healthcare13212811

**Published:** 2025-11-05

**Authors:** Hagyeong Ryu, Myungeun Lee, Soo-hyung Kim, Ju Han Kim, Hyung-jeong Yang

**Affiliations:** 1 Department of Artificial Intelligence Convergence, Chonnam National University, Buk-gu, Gwangju 61186, Republic of Korea; ryuh0627@jnu.ac.kr (H.R.);; 2 Hyper-Wide Federated Medical AI Research Center, Chonnam National University, Buk-gu, Gwangju 61186, Republic of Korea; 3 Department of Cardiology, Chonnam National University Hospital, Dong-gu, Gwangju 61469, Republic of Korea; 4 Department of Cardiology, Chonnam National University Medical School, Dong-gu, Gwangju 61469, Republic of Korea

**Keywords:** federated learning (FL), cardiovascular disease (CVD), electrocardiogram (ECG), photoplethysmography (PPG), phonocardiogram (PCG), electronic health record (EHR)

## Abstract

Federated Learning (FL) has emerged as a promising framework for multi-institutional medical artificial intelligence, enabling collaborative model development while preserving data privacy and security. Despite increasing research on federated approaches for cardiovascular disease prediction, previous reviews have largely focused on disease-specific perspectives without systematically comparing data modalities. This study comprehensively examines 28 representative investigations from the past five years, including 17 biosignal-based and 11 electronic health record (EHR)-based applications. Biosignal-based FL emphasizes personalized electrocardiogram (ECG) classification, mitigation of non-independent and identically distributed (Non-IID) data, and Internet of Things (IoT)-based monitoring using methods such as client clustering, asynchronous learning, and Bayesian inference. In contrast, EHR-based studies prioritize large-scale hospital collaboration, adaptive optimization, and secure aggregation through distributed frameworks. By systematically comparing methodological strategies, performance trade-offs, and clinical feasibility, this review highlights the complementary strengths of biosignal- and EHR-based approaches. Biosignal frameworks show strong potential for personalized, low-latency cardiac monitoring, whereas EHR frameworks excel in scalable and privacy-preserving decision support. Building upon the limitations of earlier reviews, this paper introduces data-type-centric design guidelines to enhance the reliability, interpretability, and clinical scalability of FL in cardiovascular diagnosis and prediction.

## 1. Introduction

Medical data are highly sensitive and heterogeneous, making direct sharing between institutions legally and ethically challenging [1,2]. Biosignals (e.g., electrocardiogram, ECG; photoplethysmography, PPG; phonocardiogram, PCG) and electronic health records (EHRs) often contain personally identifiable information (PII), underscoring the need for secure analytical frameworks [1,2]. In this context, federated learning (FL), which enables collaborative learning while keeping data local, has received growing attention in medical artificial intelligence [3]. FL ensures privacy protection and enables distributed model training across domains including medical imaging, clinical outcome prediction, biosignal analysis, and EHR-based modeling [4].

Cardiovascular diseases (CVD) are high-risk chronic conditions that remain among the leading causes of death worldwide [5,6,7]. As the population ages and lifestyles change, the prevalence of these diseases continues to rise, placing an increasing burden on healthcare systems [5,6,7,8,9,10]. Therefore, their diagnosis and management require continuous biosignal monitoring, prognostic prediction using historical EHR data, and multi-institutional collaboration, which are closely aligned with the technical characteristics of FL [1,11]. For example, ECG and PPG signals collected from wearable devices present challenges due to inter-patient variability and inter-device heterogeneity [12]. EHRs differ in recording formats and diagnostic coding systems across hospitals, requiring integrated analysis of diverse data such as medical histories, medication records, and laboratory test results [13]. These challenges reveal the limitations of centralized learning and have prompted active research into FL frameworks as an alternative [14,15,16,17]. This interdependence between multi-source data and distributed analytics positions FL as an ideal paradigm for cardiovascular research.

Previous reviews, such as that by Donkada et al., provided a comprehensive analysis of FL applications in CVD prediction, addressing technical issues including model structure, communication efficiency, and data privacy [14]. However, despite its disease-centric focus, the study lacked systematic classification of data-type heterogeneity and sensor-specific learning strategies. Similarly, Rahman et al. examined the application of FL for remote ECG data and communication infrastructure security policies [15], but did not discuss algorithmic design tailored to data type or concrete aspects of real-world clinical implementation. As such, existing reviews have primarily centered on disease-focused approaches, providing limited discussion of data-type-specific FL strategies, thereby highlighting the need for a more systematic analysis.

Therefore, this paper systematically analyzes FL application strategies for diagnostic and predictive studies using biosignals and EHRs, with a focus on CVDs. Specifically, it compares structural characteristics, application methods, technical approaches, and common challenges across data types to propose customized FL design guidelines. By addressing strategic differences not sufficiently covered in previous reviews, this work aims to enhance the practical applicability of CVD prediction and improve the scalability of medical artificial intelligence.

## 2. Materials and Methods

**Figure 1:**
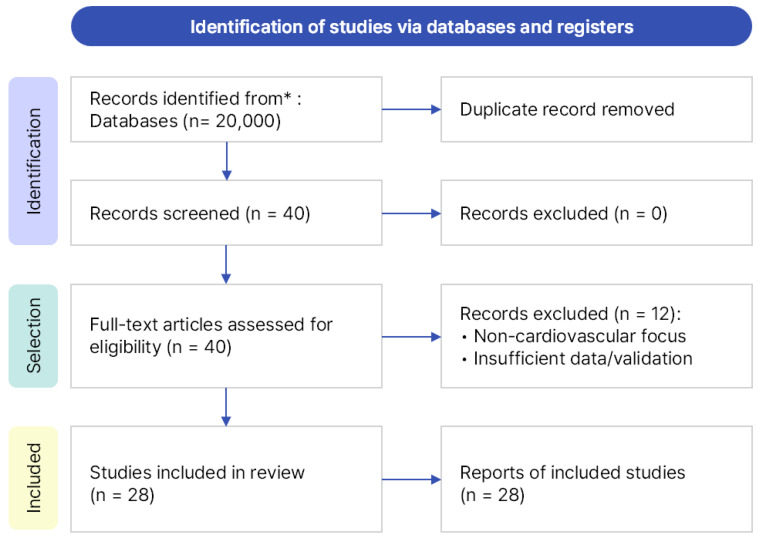
PRISMA flow diagram illustrating the process of identification, screening, eligibility assessment, and final inclusion of 28 studies. * The asterisk indicates that records were identified from major databases (Google Scholar, IEEE Xplore, and PubMed).

In this study, we conducted a literature review on federated learning (FL) for cardiovascular diseases (CVDs), covering studies published from January 2020 to March 2025. Searches were performed across three major academic databases—Google Scholar (Google LLC, Mountain View, CA, USA), IEEE Xplore (Institute of Electrical and Electronics Engineers, New York, NY, USA), and PubMed (U.S. National Library of Medicine, Bethesda, MD, USA)—using the same query and timeframe. Google Scholar was selected as the primary database because it provides broad interdisciplinary coverage across both engineering and medical research domains, enabling a unified and reproducible search process. IEEE Xplore and PubMed were additionally searched to ensure completeness; however, no new studies meeting the predefined inclusion criteria were identified. The core search query was “federated learning for cardiovascular disease”, supplemented with domain-specific keywords such as ECG, PPG, PCG, and EHR to broaden the scope. A total of 40 papers, all written in English, were identified. After manually removing duplicates and screening titles and abstracts, 28 studies were finally included for analysis. The overall search and selection process followed the PRISMA guidelines and is summarized in Figure 1.

The inclusion criteria required studies to be directly related to the prediction or diagnosis of CVD and to apply FL to real-world data sources such as biosignals and electronic health records, including medical imaging datasets when clinically relevant. Exclusion criteria included generic FL theory papers without direct CVD relevance, studies with severely limited datasets or insufficient validation, and those describing IoT/edge simulations or blockchain integrations lacking clinical applicability. While excluded from the main comparative analysis, some of these excluded studies were retained as contextual discussion. The final 28 studies were categorized into six domains: EHR-based studies, biosignal-based studies (ECG, PPG, PCG), medical imaging-based studies, IoT and edge-based studies, FL algorithmic and architectural proposals, and integrative studies. Among these, this review provides an in-depth comparative analysis of 11 EHR-based and 17 biosignal-based studies, whereas the remaining categories were referenced contextually due to their limited representation in the literature.

Previous reviews have broadly addressed both the medical applicability and technical advantages of FL [14,15]. Donkada et al. [14] conducted a scoping review summarizing the promises and challenges of FL for CVD detection. However, their analysis remained largely descriptive and did not differentiate between data modalities. Rahman et al. [15] focused on challenges and potential solutions for applying FL in cardiology, emphasizing issues such as data heterogeneity, privacy, and governance, yet maintained a disease-centric perspective without a systematic comparison of data-driven strategies. Most prior reviews remained disease-centric, with limited comparative analysis of strategy differences across data types.

**Figure 2:**
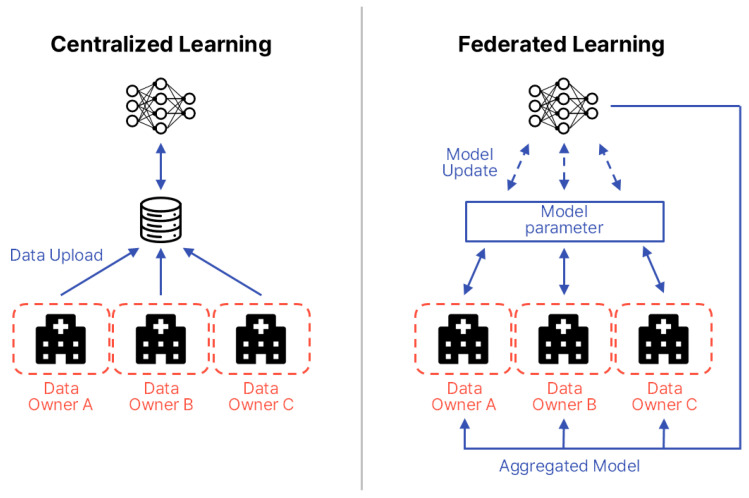
Structural comparison between centralized learning and FL. The diagram is redrawn by the authors based on Intel Community [19].

To address these gaps, this paper compares and analyzes FL application strategies for biosignals and EHRs, proposing tailored design guidelines for each data type. FL is a distributed machine learning approach in which each participating institution or device (clients) trains a model locally without transmitting raw data to a central server. Instead, only model parameters are shared. First introduced by McMahan et al. in 2016, FL has attracted significant attention as a means of simultaneously ensuring data privacy and communication efficiency [18]. In the medical domain, FL is particularly valued for enabling multi-institutional collaboration while safeguarding sensitive patient information. Figure 2 illustrates the structural differences between centralized learning and FL. In FL, the common aspect across participating data centers is the local storage and use of sensitive patient data, which is never transferred. Differences arise from variations in data distributions and modalities across institutions, including patient demographics, device types, and clinical coding standards. The information exchanged between centers and the central server is strictly limited to model parameters or aggregated updates, occasionally supplemented by performance metrics, but never raw patient data.

FL can be broadly categorized into two major architectures—Cross-Device and Cross-Silo—depending on client characteristics and scale [20]. Cross-Device FL typically involves numerous small-scale devices, such as smartphones, Internet-of-Things (IoT) sensors, and wearable devices, which contribute intermittently and often operate under constrained computational or communication resources. Cross-Silo FL, in contrast, involves a smaller number of large and relatively stable participants, such as hospitals, research institutes, and healthcare consortia, each maintaining substantial institution-level datasets.

**Figure 3:**
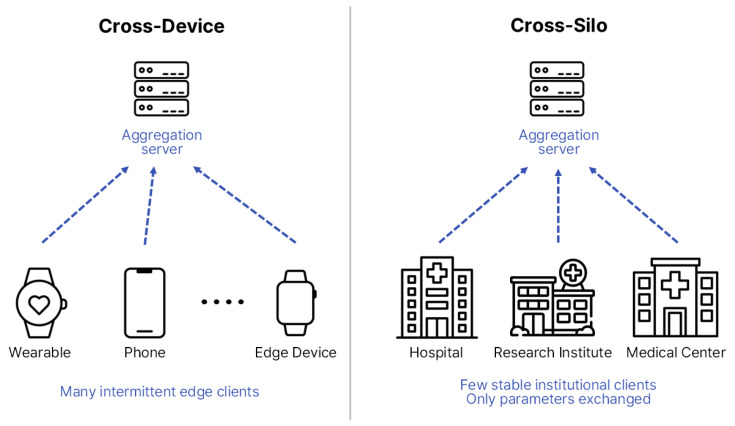
Structural comparison between Cross-Device and Cross-Silo FL structures. The diagram is redrawn by the authors based on [22], licensed under CC BY 4.0.

Figure 3 illustrates these architectural differences [21,22], showing that biosignal-based studies generally align with Cross-Device configurations owing to their decentralized and real-time nature, whereas EHR-based research is more commonly implemented in Cross-Silo settings that demand coordinated inter-institutional collaboration. Therefore, distinct structural designs and optimization strategies must be adopted according to the research objectives, data modality, and communication environment to ensure scalability, efficiency, and clinical reliability [22].

**Figure 4:**
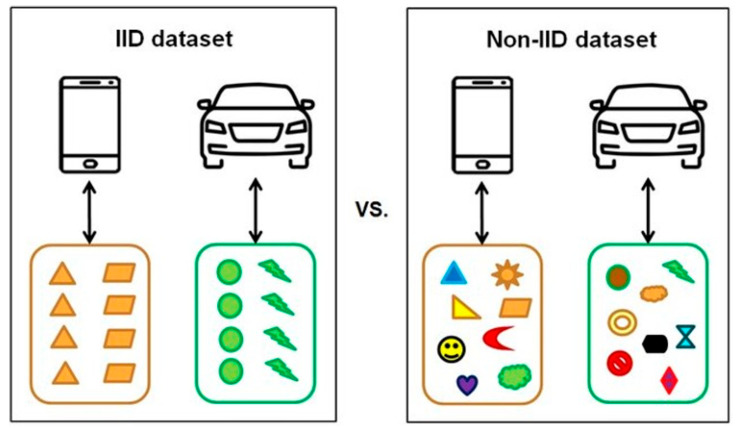
Conceptual comparison of IID vs. Non-IID datasets. Reproduced from [26], licensed under CC BY-NC-ND 4.0.

Medical data often exhibit non-independent and identically distributed (Non-IID) characteristics due to factors such as patient heterogeneity, rarity of specific diseases, and variability in equipment [23]. These challenges can lead to slower model convergence, imbalanced performance across clients, and overfitting to particular participants, ultimately limiting the practicality of medical artificial intelligence (AI) applications [24]. To overcome these issues, strategies such as personalized learning, client clustering, and importance-weighted aggregation have been proposed to mitigate data bias and enhance learning stability [23,24,25]. Figure 4 conceptually and visually illustrates the differences between independent and identically distributed (IID) and Non-IID datasets [26], emphasizing the inherent imbalance and highlighting the necessity of federated-learning frame works tailored to the unique characteristics of medical data.

## 3. Results

### 3.1. Biosignal-Based FL Applications

Table 1 summarizes FL studies employing diverse biosignals, including ECG, PCG, and PPG. Each study implemented tailored strategies based on data structure and clinical requirements. ECG-based research focused on personalization, privacy preservation, communication efficiency, and disease-specific optimization, whereas PCG- and PPG-based studies emphasized scalability in real-world clinical environments, addressing issues such as label inconsistency, regression-based blood-pressure estimation, and inter-device heterogeneity. Collectively, these studies demonstrate the diversity of federated approaches across biosignal modalities and lay the foundation for a more detailed examination of ECG, PCG, and PPG applications in the following sections.

Among these biosignals, the ECG is the most widely utilized modality for the diagnosis of CVD and is continuously collected in both clinical and wearable-device environments, making it particularly well-suited for FL applications [44]. Owing to its prevalence and diagnostic importance, recent studies have applied FL to ECG data for a range of objectives, including enhancing personalization, preserving data privacy, and enabling large-scale multi-institutional collaboration.

**Figure 5:**
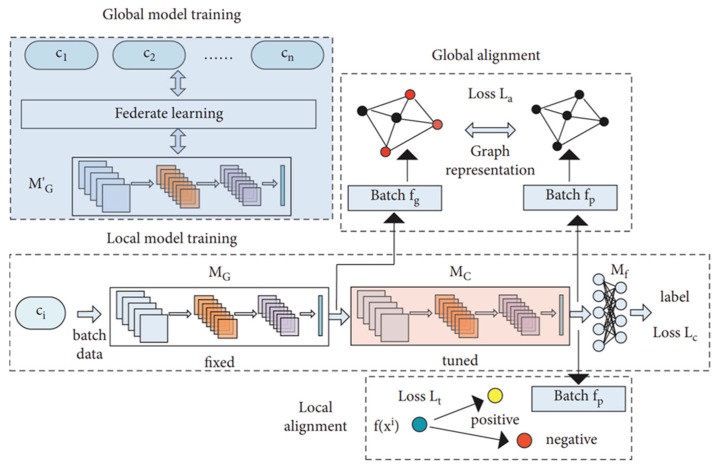
Personalized FL framework for ECG classification. Reproduced from [27], licensed under CC BY 4.0. (Note: the original figure contains the term ‘Federate learning,’ which should read as ‘Federated learning’).

Because ECG signals exhibit substantial inter-patient variability, a single global model often struggles to achieve sufficient generalization across clients. To address this limitation, Tang et al. [27] proposed a feature alignment strategy designed to harmonize ECG waveform characteristics among clients. In their empirical evaluation, the method achieved an average classification accuracy of 87.85% on local data and 83.92% on global data, outperforming existing approaches. Furthermore, classification performance was improved through the integration of graph representation learning and customized loss functions within a dual learning framework that combined global and local models. In this framework, ${M^{\prime }}_{G}$ represents the updated global model aggregated on the server, while $M_{G}$ denotes its fixed copy inherited by each client for local training. $M_{C}$ serves as the local feature extractor, and $M_{f}$ as the final classifier. The three loss terms—$L_{c}$, $L_{a}$, and $L_{t}$—optimize classification, global alignment, and local alignment, respectively. Figure 5 illustrates this personalized ECG-based FL framework, demonstrating enhanced performance through the mitigation of patient-specific variability.

ECG data in FL environments often exhibit significant Non-IID characteristics across patients, devices, and institutions, which can result in performance degradation and unstable convergence in standard aggregation algorithms such as FedAvg [18], particularly under class-imbalance conditions. To address these issues, several studies have investigated client-clustering approaches based on feature similarity to enhance personalization in Cross-Device settings. Lin et al. [28] demonstrated that their federated framework employing client clustering attained an average accuracy of 89.26% while a centralized baseline achieved 96.94% accuracy. Although the federated model performed below the centralized baseline, it enabled privacy-preserving collaborative training without sharing raw ECG data, highlighting the practical feasibility of client-clustering strategies for personalized cardiac diagnosis.

In addition to clustering-based strategies, other studies have explored optimization approaches to address the challenges posed by Non-IID ECG data. The FedGE algorithm [29] achieved an F1-score of 0.70 under Non-IID conditions, representing a 75% improvement over the FedAvg baseline, and demonstrated up to 33% faster convergence in IID settings. Similarly, a weighted FL approach [30] attained 98% accuracy, 99% sensitivity, and 91% specificity, outperforming both the standard FL configuration and centralized learning baselines in mitigating class imbalance. These results collectively highlight the effectiveness of personalized aggregation and weighting strategies for improving learning stability and fairness in heterogeneous ECG environments.

Continuous physiological indicators such as heart rate exhibit considerable variability and subject-specific uncertainty, making accurate regression prediction particularly challenging. To address this challenge, Fang et al. [31] proposed a personalized FL framework that integrates Bayesian inference to account for client-specific uncertainty, handle ECG waveform variability, and improve regression prediction accuracy. The proposed model achieved mean training/testing mean squared errors (MSEs) of 3.11/3.08 and 2.81/2.95, respectively, outperforming the FedAvg baseline (3.27/3.23).

Medical data sharing across countries and institutions is frequently constrained by legal and ethical considerations, which limits the generalizability of models trained on single-institution datasets—particularly for rare diseases such as hypertrophic cardiomyopathy (HCM). To overcome these challenges, several studies have investigated Cross-Silo FL, explainable artificial intelligence (XAI), and context-aware frameworks aimed at improving diagnostic accuracy while maintaining data privacy. Goto et al. [32] developed ECG and echocardiography models for diagnosing HCM without requiring direct data exchange between institutions in a multi-institutional collaboration. Their Cross-Silo FL models achieved C-statistics ranging from 0.90 to 0.96 across all participating sites, whereas centralized models trained locally showed AUROCs of 0.88–0.93 for internal validation but declined to 0.79–0.82 on external datasets. These results indicate that, while centralized approaches suffer from limited generalizability, FL maintained robust diagnostic performance across multinational institutions, including those in the United States and Japan.

Raza et al. [33] integrated XAI techniques with Gradient-weighted Class Activation Mapping (Grad-CAM)-based visualization to improve interpretability and diagnostic reliability for medical practitioners in real-time ECG monitoring. The proposed system achieved accuracies of 98.9% on clean data and 94.5% on noisy datasets, along with near-zero mean absolute deviation (MAD) and an 8.2% reduction in communication cost via selective layer transmission. Although the study did not directly compare its results to a centralized baseline, the reported performance was comparable to previously published centralized models (≈96.9–98.8%), demonstrating that federated approaches can simultaneously achieve privacy preservation and interpretability.

Ogbuabor et al. [34] introduced a privacy-preserving, context-aware FL framework for cardiac health monitoring that leverages both physiological and activity signals. When evaluated on an independent dataset, the framework achieved classification accuracies of 89% with a support vector machine (SVM) and 81% with a logistic regression model. The FL-based framework produced more consistent results, underscoring its advantage in both model generalization and data privacy protection while conventional centralized models exhibited poor generalization with accuracies ranging from 53% to 88% across client-level evaluations.

**Figure 6:**
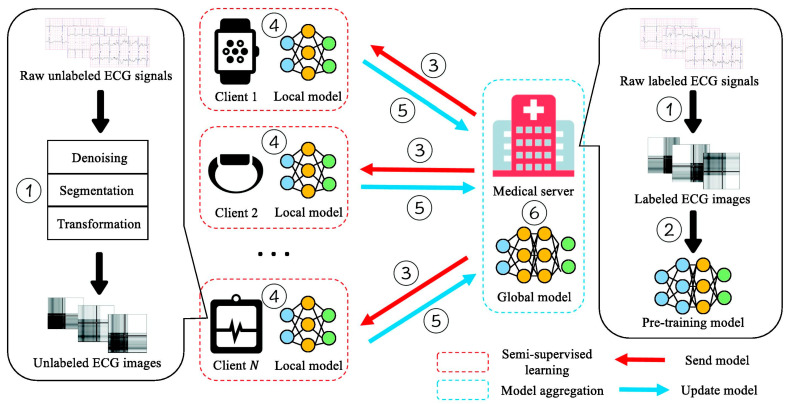
Semi-supervised FL framework for ECG anomaly prediction. Reproduced from [35], licensed under CC BY-NC-ND 4.0.

Although large amounts of ECG data are continuously collected, a substantial portion remains unlabeled because manual annotation requires both time and clinical expertise. This limitation hinders the development of accurate FL models, particularly in small or resource-limited institutions. To mitigate the shortage of labeled data, Ying et al. [35] proposed a semi-supervised FL framework termed FedECG. In this approach, a global model is first pre-trained on the central server using labeled ECG samples and then distributed to clients, where local models learn from unlabeled data before contributing updates to the server. In their experiments, they achieved 94.8% accuracy using only 50% labeled data, comparable to the 95.9% accuracy obtained from a fully supervised centralized baseline demonstrating strong robustness in limited-data settings while alleviating annotation inconsistencies across institutions. Figure 6 illustrates the overall workflow proposed by Ying et al. [35], which visualizes the semi-supervised federated training process and its interaction between the central server and local clients.

In Internet of Things (IoT) and Internet of Medical Things (IoMT) environments, ECG signals are continuously collected through wearable and mobile devices; however, centralized data processing introduces privacy risks and communication latency, making it unsuitable for real-time clinical monitoring. To address these challenges, Wang et al. [36] proposed a privacy-centric FL framework designed to protect ECG data collected in IoMT systems while maintaining diagnostic accuracy. When evaluated on the MIT-BIH dataset, the framework achieved approximately 90% accuracy for local models and global accuracies of 90.9%, 84.7%, and 78.3% under privacy budgets (ε = 1.0, 0.8, and 0.6, respectively), while reducing the probability of successful data reconstruction by nearly 50%.

Mehta and Kundra [37] implemented a lightweight adaptive FL framework for distributed edge devices in IoT-based sensor environments. Their system achieved 97.8% accuracy, 98.2% recall, and an F1-score of 97.3% for arrhythmia detection, while reducing communication latency by 35% compared with the centralized baseline. These findings demonstrate the practicality of deploying FL in real-time cardiac health monitoring systems and emphasize the role of differential privacy (ε = 1.5) in safeguarding sensitive physiological data.

In medical FL scenarios, heterogeneity in network bandwidth, computational capacity, and data distribution across clients often leads to inconsistent training performance and latency issues, rendering synchronous FL inefficient due to the so-called straggler problem. To address this limitation, Sakib et al. [38] proposed an asynchronous FL architecture that accommodates differences in communication delay and computational resources among medical clients, maintaining convergence stability and predictive accuracy. In their experiments on arrhythmia detection, the asynchronous model achieved approximately 95% accuracy after convergence, demonstrating higher area under the curve (AUC), faster execution, and lower memory consumption compared with the synchronous baseline. Similarly, Khan et al. [39] applied an asynchronous framework to CVD prediction, achieving accuracies of 89.1% and 89.9% on two independent datasets—surpassing the synchronous counterpart while substantially improving training efficiency. Collectively, these studies highlight the potential of asynchronous FL to enhance scalability and responsiveness in heterogeneous medical environments.

Congestive heart failure (CHF) is a progressive cardiac disorder in which early detection plays a vital role in preventing disease progression and improving patient outcomes. However, data scarcity and strict privacy regulations often limit large-scale model development in this domain. To overcome these challenges, Zou et al. [40] applied a convolutional neural network (CNN)-based UNet++ architecture within a FL framework, utilizing distributed ECG data to construct a high-performance predictive model for early CHF detection. Their approach processed RR interval–based ECG signals through the UNet++ architecture and integrated them into a multi-institutional FL setting, enabling collaborative model training without direct data sharing. The study reported accuracy of 89.83% for the centralized baseline and 87.54% for the FL configuration, outperforming other UNet++ variants while preserving data privacy and demonstrating the feasibility of FL for early cardiac risk prediction.

Beyond ECG data, other biosignals such as PCG and PPG have also been investigated in FL applications, each requiring approaches tailored to their distinct physiological characteristics and data modalities. PCG, derived from the acoustic signals generated by cardiac mechanical activity, serves as a widely used non-invasive and cost-effective modality for CVD screening, particularly in detecting abnormal heart sounds [45]. However, in multi-institutional settings, challenges such as data fragmentation, inconsistent labeling, and privacy restrictions limit the feasibility of centralized learning. To address these challenges, Qiu et al. [41] developed a series of FL models using PCG data, demonstrating that the federated paradigm can be effectively applied to heart-sound classification with FedAvg-based architectures such as Fed-MLP, Fed-CNN1, and Fed-CNN2. In their experiments, the centralized CNN2 model achieved the highest accuracy of 76.2%, whereas its federated counterpart (Fed-CNN2) achieved 72.1% under independent and identically distributed (IID) and 65.4% under Non-IID settings with global model aggregation. Although the FL model exhibited a modest accuracy reduction relative to the centralized baseline, it achieved the best sensitivity (59.2%) and specificity (65.9%), validating the feasibility of FL for PCG-based cardiac sound classification across distributed institutions.

Qiu et al. [42] further proposed the Fed-MStacking approach—an ensemble technique that integrates heterogeneous local models, including random forest (RF), feedforward neural networks (FNN), and convolutional neural networks (CNN). This framework was designed to mitigate label inconsistencies and misalignments among institutions participating in PCG-based FL. Experimental results showed that Fed-MStacking produced more stable and higher performance after data balancing, achieving an unweighted average recall (UAR) of 79.31% on coronary artery disease (CAD) datasets, outperforming homogeneous stacking (75.18%) and existing baseline methods (71.86%). These findings highlight the robustness and adaptability of ensemble-based FL strategies in heterogeneous PCG environments, emphasizing their potential to enhance generalization across diverse healthcare institutions.

PPG is a non-invasive optical biosignal that measures volumetric changes in blood flow and is widely employed for continuous cardiovascular monitoring [46], including blood pressure (BP) estimation. However, variations in device calibration, nonlinear signal–BP relationships, and privacy concerns in multi-device environments hinder the feasibility of centralized training. To overcome these limitations, Brophy et al. [43] proposed an FL framework that integrates generative adversarial networks (GANs) for PPG-based BP prediction. In this approach, each client trains a local GAN model using its own calibrated PPG data, and the server aggregates model parameters to construct a global predictor without requiring direct data sharing. Experimental results demonstrated that the FL-based model achieved a mean arterial pressure (MAP) error of 2.95 mmHg and a root mean square error (RMSE) of 0.24—only slightly higher than the centralized baseline (RMSE 0.19)—indicating that the federated model preserved near-equivalent predictive performance while ensuring privacy. Although the MAP value itself cannot be directly interpreted under the Association for the Advancement of Medical Instrumentation (AAMI)/American National Standards Institute (ANSI)/International Organization for Standardization (ISO) criteria (which apply to systolic and diastolic blood pressure), these results show that the proposed FL framework effectively addresses the nonlinear nature of time-series signals and inter-device heterogeneity, thereby improving regression prediction performance by accounting for individual differences.

**Figure 7:**
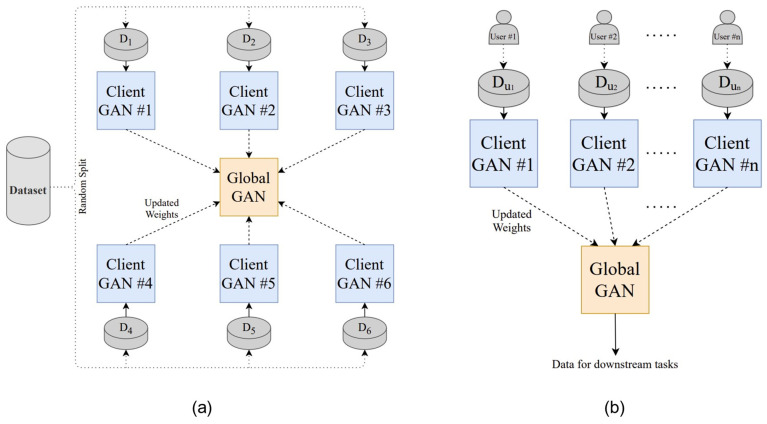
FL architecture for PPG-based blood pressure estimation: (**a**) GAN-based FL model structure; (**b**) system implementation and transmission flow. Reproduced from [43], licensed under CC BY 4.0.

Figure 7 illustrates the FL framework for PPG-based BP estimation, with (a) showing the GAN-based FL model architecture and (b) depicting the system implementation and data-transmission flow. These findings demonstrate the enhanced clinical applicability of wearable-based real-time blood-pressure estimation and highlight the potential of FL–GAN integration for privacy-preserving cardiovascular monitoring.

### 3.2. EHR-Based FL Applications

EHRs contain extensive clinical information, including diagnostic codes, medication histories, and laboratory test results, making them an essential foundation for medical artificial intelligence (AI) research. However, inter-hospital heterogeneity and the sensitive nature of personal health information raise significant challenges for centralized model development. FL offers a promising alternative by enabling collaborative training without the need to share raw EHR data.

As summarized in Table 2, EHRs provide a robust foundation for FL by enabling collaborative model development while ensuring data privacy through local storage. Because of their high dimensionality and often unstructured format—including diagnostic codes, medication histories, and laboratory test results—EHRs demand FL strategies capable of efficiently processing complex and heterogeneous clinical data [47].

Early studies demonstrated the feasibility of applying distributed learning to EHRs for CVD prediction. For example, Kavitha Bharathi et al. [48] implemented basic FL models using deep learning and conventional classifiers such as logistic regression (LR) and support vector machines (SVM), achieving accuracies of 82.38% and 90.3%, respectively, compared with a 95.8% centralized baseline. Similarly, Sharma and Sharma [49] trained a convolutional neural network (CNN)-based FL framework that achieved 94.99% accuracy—closely approximating the 97% accuracy of its centralized counterpart—demonstrating the ability of FL to preserve both privacy and predictive performance. Furthermore, Ramaswami [50] compared multiple classifiers in an FL setting, reporting diagnostic performance metrics between 0.95 and 0.96 for accuracy, precision, recall, and F1-score, outperforming other privacy-preserving and conventional approaches. Collectively, these studies confirm that FL can achieve diagnostic accuracy comparable to centralized learning while ensuring data confidentiality, thus validating its applicability for real-world EHR-based cardiovascular prediction.

Because EHRs contain sensitive personal information, even FL frameworks can face security and privacy risks such as model inversion or information leakage. To mitigate these threats, recent studies have focused on security-centric FL designs that incorporate privacy-preserving mechanisms and decentralized architectures.

Lee et al. [51] proposed a sequential pattern-mining approach integrated with FL to securely extract disease-related patterns from distributed EHR data. In their framework, differential privacy (DP) and secure aggregation were implemented to protect sensitive patient information during model updates, enabling peer-to-peer (P2P) sharing of predictive rules without direct data exchange. The study reported only minor performance trade-offs when DP was applied, and aggregated models maintained stable F1-scores and area under the curve (AUC) values even as the number of data partitions increased, demonstrating the scalability of privacy-preserving EHR analysis.

Building on this concept, Wei et al. [52] introduced a fully decentralized online FL architecture named DeFedHDP to eliminate single points of failure and enhance robustness in multi-institutional environments. The design employed Gaussian-noise–based differential privacy and a one-point bandit feedback (OPBF) technique to prevent gradient vanishing. The system achieved approximately 90% accuracy, with all clients recording AUCs above 0.93 and F1-scores exceeding 0.90, while showing faster runtime and improved communication efficiency compared with homomorphic encryption–based methods. These findings collectively underscore the growing importance of decentralized and privacy-preserving FL frameworks for securing sensitive EHR data in clinical applications.

In many clinical settings—particularly those relying on Internet of Things (IoT)–based devices or operating with limited infrastructure—the deployment of conventional FL frameworks remains challenging. EHR data collected from diverse sources such as wearable sensors are highly heterogeneous, while privacy, bandwidth, and computational constraints further complicate distributed learning. To address these limitations, several studies have explored lightweight and adaptive FL approaches designed for resource-constrained environments. Jalal et al. [53] proposed a horizontal FL (HFL) framework combined with the random forest (RF) algorithm to enable heart disease prediction using multi-institutional EHR data. Their experiments demonstrated that the HFL-RF model achieved up to 97.22% accuracy and an F1-score of 96%, improving by approximately 7.1% over the centralized baseline (~85% accuracy). These results highlight the potential of FL to enhance accessibility and expand its applicability in low-resource healthcare environments.

**Figure 8:**
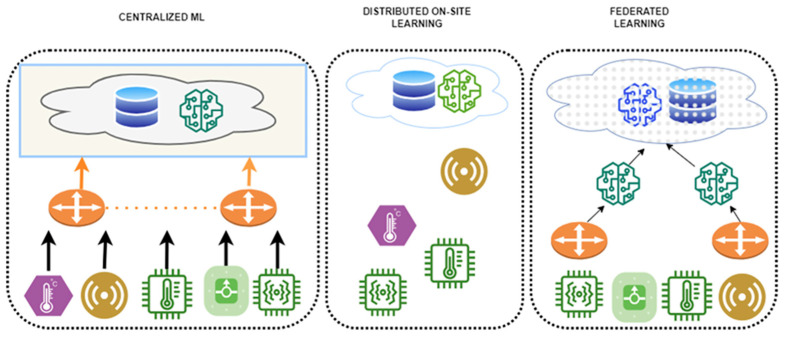
System architecture of a clustering-based FL framework utilizing IoT-based EHR data. Reproduced from [54]. licensed under CC BY 4.0.

Bebortta et al. [54] extended EHR-based FL to IoT-integrated medical ecosystems by introducing FedEHR, a clustering-based hierarchical FL framework that processes heterogeneous sensor-derived EHR data. This structure ensured both communication efficiency and predictive accuracy by reflecting variations in data distribution and device characteristics. The FedEHR model achieved a peak accuracy of 99.86%, surpassing the centralized SVM baseline (≈95–96%) and outperforming all other benchmark models. It also demonstrated faster convergence and greater efficiency in communication volume and computational cost. Figure 8 depicts this hierarchical FL structure within an IoT environment, illustrating the process by which data are trained locally on wearable devices and subsequently aggregated on the server.

The coronary artery calcification score (CACS) is a clinically significant biomarker for assessing coronary artery disease (CAD) risk; however, its measurement via computed tomography (CT) is expensive and exposes patients to radiation. Developing accurate prediction models can reduce unnecessary imaging, yet limited data availability and stringent privacy regulations often restrict inter-hospital data sharing. FL offers a practical solution by enabling collaborative model training without the need to exchange sensitive patient information.

**Figure 9:**
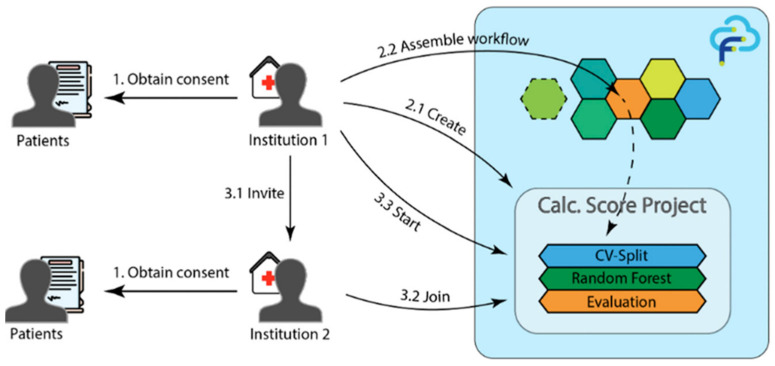
FL system architecture using the FeatureCloud platform. Reproduced from [55], licensed under CC BY 4.0.

To address this issue, Wolff et al. [55] developed a distributed learning framework using the FeatureCloud platform for predicting CACS in multi-institutional settings. The framework was designed to ensure privacy through consent-based participation and secure model-parameter exchange among hospitals. The FL model achieved an accuracy of 67.65%, sensitivity of 66.67%, and specificity of 68.57%, which were comparable to the centralized baseline (accuracy 67.65%, area under the curve [AUC] 0.755). Figure 9 illustrates the FeatureCloud architecture, emphasizing how privacy and compliance are maintained across institutions during federated training. These results demonstrate that FL can facilitate privacy-preserving collaboration for cardiovascular risk prediction in real-world multi-center environments.

The effective management of chronic diseases such as CVD increasingly depends on Internet of Things (IoT)–enabled wearable devices that continuously generate real-time EHR data. However, IoT environments frequently experience data-quality degradation, network instability, and data drift, which complicate federated model training. To overcome these challenges, Birari et al. [56] proposed an adaptive FL framework integrating IoT data streams with adaptive gradient clipping (AGC) to stabilize training and reduce communication overhead. Their model, termed FTL-AGC, achieved an average area under the curve (AUC) of 88.5%—the highest among benchmark models—demonstrating robustness despite the absence of direct centralized baseline comparisons.

In another study on heart disease and stroke prediction, Potti et al. [57] implemented a server–client FL framework and directly compared it with centralized baselines using the same dataset split. The best centralized model, based on the random forest (RF) algorithm, achieved accuracy, whereas their FL implementation attained 96.3% accuracy and an F1-score of 91.2%. These results highlight the potential of FL to achieve superior predictive performance while maintaining patient data privacy.

Kapila et al. [58] proposed a hybrid approach that combines feature selection and feature extraction techniques to enhance FL performance on high-dimensional EHR data. Diagnostic codes and medication histories were first filtered using analysis of variance (ANOVA) and Chi-square tests, and the reduced feature space was then processed via linear discriminant analysis (LDA), improving both learning efficiency and predictive reliability. The method achieved 88.52% accuracy and an F1-score of 89.23% on the Cleveland heart disease dataset, outperforming both the FL-only baseline and conventional machine-learning methods. Although no centralized baseline was reported, this study demonstrated the advantages of integrating dimensionality-reduction techniques within FL frameworks for improved clinical decision support.

### 3.3. Comparative Analysis of Biosignal- and EHR-Based FL

This section provides a comparative analysis of the characteristics, technical challenges, applied methodologies, and representative applications of biosignal- and EHR-based FL. As summarized in Table 3, biosignals are high-resolution, real-time time-series data that are well suited for personalized modeling and deployment in resource-constrained or mobile environments. In contrast, EHR data comprise both structured and unstructured records, making them more appropriate for inter-hospital collaboration, large-scale system integration, and longitudinal disease management. These distinctions form the basis for the following discussion, which explores how the inherent differences between biosignal and EHR modalities influence FL design strategies, optimization approaches, and clinical applicability.

In addition to the comparative characteristics discussed above, it is also essential to examine the publicly available datasets that have facilitated FL research across both biosignal and EHR domains. Table 4 summarizes representative open datasets commonly used in the literature, encompassing modalities such as ECG, RR intervals, heart sounds, and structured clinical records. Although these public datasets are relatively limited in size compared with production-scale clinical data, FL remains indispensable as it enables the integration of data from multiple institutions and devices without compromising patient privacy.

In practice, individual datasets are frequently fragmented across institutions, each containing a limited number of samples. Through distributed computation, FL allows these fragmented datasets to be jointly leveraged, thereby increasing statistical power, improving model generalizability, and overcoming privacy constraints that hinder centralized data pooling. Consequently, even when single datasets appear small, FL provides a robust mechanism to bridge institutional data silos and approximate large-scale, demographically and clinically representative cohorts suitable for real-world medical AI applications.

## 4. Discussion

Building on the comparative analysis presented in Section 3.3, it is evident that FL provides unique advantages for both biosignal-based and EHR-based applications in CVD diagnosis while simultaneously encountering shared technical challenges.

Biosignal-based FL is optimized for personalized diagnosis and real-time physiological monitoring. Continuous time-series signals—such as ECG, PPG, and PCG—collected through wearable or Internet of Things (IoT) devices are widely applied to the early detection of arrhythmia, heart failure, hypertension, and other cardiovascular conditions. Nevertheless, persistent challenges remain, including Non-IID data distributions, limited labeled datasets, and sensor heterogeneity. Recent approaches have employed client clustering, personalized model training, asynchronous learning, Bayesian inference, and generative adversarial network (GAN)–based data augmentation to mitigate these issues. The incorporation of explainable artificial intelligence (XAI) techniques has further enhanced interpretability, while multi-institutional FL frameworks have improved clinical reliability and cross-site generalizability.

In contrast, EHR-based FL is better suited for large-scale collaboration across medical institutions and longitudinal disease management. EHR data—comprising both structured variables and unstructured clinical narratives—are characterized by inter-hospital heterogeneity, inconsistent data standards, and stringent privacy constraints. To address these issues, techniques such as feature selection and extraction, fully decentralized learning, time-series pattern mining, and IoT integration have been adopted. These methods have demonstrated effectiveness in real-world applications, including stroke-risk prediction, coronary artery calcium-score estimation, and chronic heart-disease management, underscoring the complementary strengths of biosignal- and EHR-oriented FL approaches in precision cardiology.

Despite their complementary strengths, both biosignal-based and EHR-based FL approaches continue to face shared challenges in privacy protection, model interpretability, communication efficiency, and learning stability. Biosignal-oriented FL has proven particularly effective in resource-constrained and personalized monitoring scenarios, whereas EHR-based FL offers advantages in long-term inter-institutional collaboration and large-scale system integration. Nevertheless, persistent technical limitations—such as Non-IID data distributions, label imbalance, communication-resource constraints, and potential security vulnerabilities—require continuous methodological innovation.

From a systems perspective, scalability and communication cost remain critical determinants of performance. In Cross-Device settings (e.g., wearable or mobile devices), lightweight architectures, model-compression techniques, and asynchronous aggregation can reduce per-round transmission volume and end-to-end latency, although these optimizations may demand additional communication rounds to achieve convergence. In Cross-Silo environments (e.g., hospitals or research networks), larger models trained through synchronous rounds and scheduled client participation enhance model stability and reproducibility but often incur higher per-round communication overhead. These design choices reflect inherent trade-offs among communication efficiency, convergence rate, and on-Silo computational capacity, all of which must be carefully balanced to meet deployment constraints and clinical latency requirements in real-world medical settings.

Beyond the technical challenges discussed above, FL in cardiology must also address critical privacy and security vulnerabilities. Common threats include membership inference, gradient leakage or inversion, model inversion, and data poisoning attacks, each of which may expose sensitive patient information or compromise the integrity of collaborative model training [36]. To mitigate these risks, several privacy-preserving strategies have been developed, including secure aggregation, differential privacy (DP) with tunable ε-budgets to balance privacy–utility trade-offs, homomorphic encryption (HE), and trusted execution environments (TEEs) [15].

Recent research on decentralized FL frameworks has further explored the trade-offs between DP and HE under realistic computational and communication constraints, emphasizing that the choice of privacy mechanism must align with the resource availability and latency tolerance of the target environment [52]. In a broader context, prior surveys have delineated the structural and regulatory differences between Cross-Device FL (e.g., wearable sensors and mobile devices) and Cross-Silo FL (e.g., hospitals and institutional networks) [59]. The former typically emphasizes lightweight differential privacy and asynchronous aggregation for efficiency, whereas the latter relies on secure aggregation and homomorphic encryption to satisfy stricter scalability, governance, and compliance requirements.

It is crucial to contextualize FL performance relative to traditional centralized models. Across multiple studies, FL has consistently demonstrated performance comparable to centralized training under identical data partitions, typically exhibiting only marginal differences in metrics such as area under the curve (AUC) or root mean square error (RMSE). Unlike centralized approaches, FL preserves patient privacy while enabling collaborative learning across institutions and devices, thereby underscoring its distinct clinical value. However, the present analysis also reveals critical limitations in the datasets currently used for medical FL research. Most biosignal and EHR datasets remain small in scale, demographically imbalanced, and often lack external validation. For instance, the frequent reliance on UCI tabular datasets does not capture the complexity or heterogeneity of real-world, production-scale EHR systems. Furthermore, many studies rely on single-site internal validation or omit essential preprocessing details, raising concerns regarding reproducibility and methodological rigor.

To address these challenges, future research should prioritize the enhancement of dataset representativeness and standardization in FL. Building multi-ethnic and multi-institutional cohorts will improve demographic and institutional diversity, while adopting harmonized clinical coding systems—such as SNOMED CT [60] and Logical Observation Identifiers Names and Codes (LOINC) [61]—can strengthen interoperability across healthcare sites. Additionally, incorporating device metadata and drift labeling for biosignal recordings will facilitate the capture of longitudinal variability. The development of publicly reusable FL benchmark datasets with standardized data splits and comprehensive documentation will promote transparency, reproducibility, and cross-study comparability. These initiatives are closely aligned with emerging benchmark frameworks such as MedPerf [62] and LEAF [63], which aim to foster standardized, reproducible evaluation practices in FL research. These efforts will establish more robust, diverse, and ethically responsible foundations for future FL-based cardiovascular research. Moreover, these issues underscore the need for stronger critical appraisal in future FL studies—particularly through transparent reporting of preprocessing, open access to code and data when feasible, and the use of external or prospective validation to ensure methodological quality and reproducibility.

Beyond methodological considerations, the clinical deployment of FL in cardiology must comply with international healthcare regulations such as the Health Insurance Portability and Accountability Act (HIPAA) and the General Data Protection Regulation (GDPR). These frameworks mandate the secure management of protected health information (PHI), comprehensive risk assessment, and adherence to medical software life-cycle standards. Ensuring safe deployment further requires external or prospective validation and continuous monitoring for dataset or site drift to maintain robustness across diverse populations and healthcare institutions [64]. Moreover, tools such as model cards, audit trails, and human-in-the-loop review mechanisms can strengthen transparency, traceability, and accountability throughout the clinical adoption process. These regulatory and practical imperatives are exemplified by federated use cases such as the multinational hypertrophic cardiomyopathy (HCM) diagnosis study [32] and the FeatureCloud platform for coronary artery disease (CAD) risk prediction [55], both of which address privacy, governance, and compliance challenges inherent to cross-border medical collaboration.

This review is subject to certain limitations, primarily the lack of quantitative verification in real-world clinical applications and the absence of long-term, clinical trial-based evaluation. These limitations underscore the existing gap between experimental FL studies and clinical implementation, highlighting the urgent need for translational validation. Future research should therefore focus on developing customized FL architectures that reflect the clinical characteristics and data structures of specific disease groups. In addition, dynamic client participation strategies informed by data heterogeneity, automated data standardization and preprocessing pipelines, and the integration of explainable artificial intelligence (XAI) techniques should be prioritized. Furthermore, establishing clinically interpretable and regulatory-compliant FL systems will be essential for ensuring safe and effective deployment in healthcare environments. The establishment of continuous and self-adaptive FL environments will further enhance model robustness and scalability. Collectively, these directions are expected to serve as key strategies for improving the accuracy, reliability, and practicality of FL-based CVD diagnostic systems, thereby broadening their applicability in real-world medical environments.

## 5. Conclusions

This review comprehensively examined and compared FL application strategies for the diagnosis and prediction of cardiovascular diseases (CVDs), focusing on two primary types of medical data: biosignals and electronic health records. Unlike earlier reviews confined to specific disease groups, this study provides a systematic, data-type-oriented synthesis of technical challenges and corresponding methodological solutions, offering a multidimensional perspective on the clinical applicability, scalability, and reliability of FL in modern healthcare systems

Biosignal-based FL has been extensively applied to predict a range of cardiovascular conditions, including arrhythmia, heart failure, and hypertension, using time-series data such as ECG, PPG, and PCG signals. To overcome challenges such as Non-IID data distributions and limited communication resources, studies have adopted techniques including client clustering [28], asynchronous learning [38], personalized model training [27], and explainable artificial intelligence (XAI) [33]. These strategies have enabled wearable-based real-time monitoring and personalized diagnosis, thereby enhancing the clinical practicality of FL in precision cardiology. Conversely, EHR-based FL has utilized feature selection and extraction [58], time-series pattern mining [51], fully decentralized architectures [52], and adaptive learning [56] to manage complex structural heterogeneity and stringent privacy constraints. Such approaches have demonstrated effectiveness in domains requiring inter-institutional collaboration, such as stroke prediction, coronary artery disease (CAD) risk assessment, and chronic disease management, underscoring the complementary roles of biosignal- and EHR-oriented FL systems in modern cardiovascular medicine.

Despite their distinct advantages, both biosignal-based and EHR-based FL approaches share common challenges related to privacy protection, model interpretability, learning stability, and communication efficiency [15]. Addressing these issues is critical not only for improving model performance but also for ensuring clinical reliability, transparency, and physician trust in FL-driven decision support systems.

Future research should therefore prioritize standardized data representation, dynamic client participation mechanisms, and XAI-driven interpretability enhancement. In addition, automation of data preprocessing and the establishment of continuous, adaptive learning infrastructures are needed to sustain long-term model performance across evolving clinical environments. These advancements will facilitate multi-institutional clinical trials and benchmark standardization, ultimately improving the accuracy, reliability, and scalability of FL-based CVD diagnostic systems in real-world clinical practice.

## Figures and Tables

**Table 1:** Summary of FL research cases based on biosignals.

Biosignal	Reference	Objective	FL Strategy	Centralized	FL
ECG	[27]	Personalized ECG classification	Feature alignment, Dual model	82.6%	87.8% (local) 83.9% (global)
	[28]	Personalization enhancement	Client clustering	96.9%	89.3% (average)
	[29]	Handling Non-IID data	Local optimization	N/A	0.70 F1-score
	[30]	Handling Non-IID data	Importance weighted updates	≈95.0%	98.0%
	[31]	Heart rate regression	Bayesian inference	N/A	MSE 2.81
	[32]	Rare cardiac disease diagnosis	Cross-Silo collaboration	AUROC 0.88–0.93 (int.); 0.79–0.82 (ext.)	AUROC 0.90–0.96 (multi-site, incl. ext)
	[33]	Providing visual explanations	Explainable AI	~96.9–98.8% (prior works, indirect comparison)	98.9% (clean), 94.5% (noisy)
	[34]	Context-aware adaptation	Context-aware FL	53–88% (client-level test)	89% (SVM) 81% (LR)
	[35]	Semi-supervised learning	Semi-supervised FL	95.9% (supervised baseline)	94.8% (semi-supervised, 50% labeled)
	[36]	Privacy-centric design	IoMT-based FL	N/A	90.9%
	[37]	Remote health monitoring	Edge device optimization	N/A	97.8%
	[38]	Handling delays	Asynchronous FL	N/A	~95.0%
	[39]	Handling delays	Asynchronous FL	N/A	89.9%
	[40]	Early CHF prediction	CNN-integrated FL	89.8%	87.5%
PCG	[41]	Abnormal heart sound detection	Local training, Global update	76.2%	72.1%
	[42]	Label inconsistency	Stacking-based ensemble	75.2% UAR	79.3% UAR
PPG	[43]	Blood pressure estimation	GAN-based FL	RMSE 0.19/0.23	RMSE 0.24/0.25, MAP error 2.95 mmHg

Unless otherwise specified, reported performance values represent classification accuracy. Other metrics are explicitly stated (e.g., F1-score, RMSE, AUROC). Abbreviations: AUROC, Area Under the Receiver Operating Characteristic Curve; F1-score, F1 measure; MSE, Mean Squared Error; RMSE, Root Mean Square Error; MAP, Mean Arterial Pressure; UAR, Unweighted Average Recall; SVM, Support Vector Machine; LR, Logistic Regression; N/A, Not Applicable (Centralized baseline not reported); int., internal validation; ext., external validation.

**Table 2:** Summary of FL research cases based on EHR.

Category	Reference	Objective	FL Strategy	Centralized	FL
Basic Framework	[48]	Heart disease prediction	Basic FL, Deep learning	LR 95.8%	LR 82.4% SVM 90.3%
	[49]	Data security & accuracy	Local CNN, Server aggregation	97.0%	94.9%
	[50]	Diagnostic performance	Classifier comparison	N/A	0.95–0.96 (accuracy, precision, recall, F1-score)
Security-Centric Design	[51]	Privacy-preserving pattern mining	Sequential mining, Differential privacy	N/A	Minor loss with DP, stable F1-score, AUC
	[52]	Decentralized online learning	Fully decentralized FL, Local updates	N/A (compared qualitatively to FedAvg)	≈90.0%
Low-Resource/IoT	[53]	Low-resource FL	Horizontal FL, RF	≈85%	97.2%
	[54]	IoT-based heart disease prediction	Clustering-based FL	≈95–96%	99.8%
Multi- Institutional	[55]	Hospital collaboration	FeatureCloud FL	67.6%, AUC 75.52	67.6%, AUC 75.1
Adaptive/Advanced	[56]	Adaptive learning	Adaptive Gradient Clipping	N/A	AUC 88.5%
	[57]	Distributed learning improvement	Server–client FL	RF 93.3%	96.3%, F1 = 91.2%
	[58]	Feature selection & extraction	ANOVA, Chi-square, LDA	N/A	88.5%., F1 = 89.2%

Unless otherwise specified, reported performance values represent classification accuracy. Other metrics are explicitly stated (e.g., F1-score, RMSE, AUROC). Abbreviations: AUC, Area Under the Curve; LR, Logistic Regression; SVM, Support Vector Machine; RF, Random Forest; DP, Differential Privacy; ANOVA, Analysis of Variance; LDA, Linear Discriminant Analysis; N/A, Not Applicable (Centralized baseline not reported).

**Table 3:** Comparison of FL based on biosignals and EHR.

Category	Biosignal-Based FL	EHR-Based FL
Data Characteristics	Time-series, high-resolution, real-time collection	Mixed structured and unstructured data, medical records
Main Challenges	Non-IID, personalization, communication resources	Structural heterogeneity, standardization, privacy
Applied Techniques	XAI, asynchronous learning, lightweight models, GANs	Feature selection/extraction, distributed learning, pattern mining
Representative Applications	Arrhythmia, heart failure, real-time monitoring	Stroke, coronary artery disease, chronic disease management

**Table 4:** Representative Public Datasets.

Domain	Dataset	Modality	Reference	Sample Size	Source
Biosignal	MIT-BIH Arrhythmia Database	ECG	[28,30,33,35,36,37,38]	≈109 k beats (47 subjects)	PhysioNet
	MIT-BIH Supraventricular Arrhythmia Database	ECG	[38]	Not specified	PhysioNet
	INCART 12-lead Arrhythmia Database	ECG	[38]	Not specified	PhysioNet
	Sudden Cardiac Death Holter Database	ECG	[38]	Not specified	PhysioNet
	NSR-RR Interval Database	RR-interval	[40]	54 patients	PhysioNet
	CHF-RR Interval Database	RR-interval	[40]	29 patients	PhysioNet
	Physical Activity Recognition Dataset	ECG + Activity data	[34]	12 patients	Middlesex Univ.
	CinC Challenge 2016 Heart Sound Dataset	PCG	[41,42]	3240 samples (764 subjects)	PhysioNet
	Cuffless Blood Pressure Estimation	PPG, ECG ABP	[43]	≈144 k samples	Kaggle, UCI ML Repo.
	University of Queensland Vital Signs Dataset	PPG, ABP	[43]	900 samples	Univ. of Queensland, RAH
EHR	UCI Heart Disease Database	Structured (ECG, clinical data)	[48,49,58]	303 subjects (14 features)	UCI ML Repo.
	UCI Heart Disease Database (multi-site)	Structured (clinical data)	[53]	1190 subjects (4 hospital sites)	UCI ML Repo. (multi-site)

RAH = Royal Adelaide Hospital.

## Data Availability

No new data were created or analyzed in this study. Data sharing is not applicable to this article.

## Abbreviations

FL	Federated Learning
ECG	Electrocardiogram
PPG	Photoplethysmography
PCG	Phonocardiogram
EHR	Electronic Health Record
CVD	Cardiovascular Diseases
IoMT	Internet of Medical Things
Non-IID	Non-Independent and Identically Distributed
Cross-Device FL	Federated Learning across numerous edge or wearable devices (e.g., ECG sensors)
Cross-Silo FL	Federated Learning among a limited number of institutions (e.g., hospitals, research centers)
C-statistic	Concordance statistic; equivalent to the area under the ROC curve (AUC) used in medical evaluation
